# Metformin Pre-Treatment as a Means of Mitigating Disuse-Induced Rat Soleus Muscle Wasting

**DOI:** 10.3390/cimb45040201

**Published:** 2023-04-04

**Authors:** Timur M. Mirzoev, Inna I. Paramonova, Sergey V. Rozhkov, Ekaterina P. Kalashnikova, Svetlana P. Belova, Sergey A. Tyganov, Natalia A. Vilchinskaya, Boris S. Shenkman

**Affiliations:** Myology Laboratory, Institute of Biomedical Problems RAS, Moscow 123007, Russia

**Keywords:** hindlimb suspension, soleus muscle, metformin, AMPK, anabolic signaling, 18S+28S rRNA, ubiquitin, calpain-1

## Abstract

Currently, no ideal treatment exists to combat skeletal muscle disuse-induced atrophy and loss of strength. Because the activity of AMP-activated protein kinase (AMPK) in rat soleus muscle is suppressed at the early stages of disuse, we hypothesized that pre-treatment of rats with metformin (an AMPK activator) would exert beneficial effects on skeletal muscle during disuse. Muscle disuse was performed via hindlimb suspension (HS). Wistar rats were divided into four groups: (1) control (C), (2) control + metformin for 10 days (C+Met), (3) HS for 7 days (HS), (4) metformin treatment for 7 days before HS and during the first 3 days of 1-week HS (HS+Met). Anabolic and catabolic markers were assessed using WB and RT-PCR. Treatment with metformin partly prevented an HS-induced decrease in rat soleus weight and size of slow-twitch fibers. Metformin prevented HS-related slow-to-fast fiber transformation. Absolute soleus muscle force in the HS+Met group was increased vs. the HS group. GSK-3β (Ser9) phosphorylation was significantly increased in the HS+Met group vs. the HS group. Metformin pre-treatment partly prevented HS-induced decrease in 18S+28S rRNA content and attenuated upregulation of calpain-1 and ubiquitin. Thus, pre-treatment of rats with metformin can ameliorate disuse-induced reductions in soleus muscle weight, the diameter of slow-type fibers, and absolute muscle strength.

## 1. Introduction

It is well acknowledged that long-term physical inactivity (disuse) in the form of bed rest, limb immobilization/suspension, and prolonged exposure to microgravity triggers skeletal muscle wasting in both rodents and humans [1,2,3,4,5]. Disuse-induced skeletal muscle atrophy is characterized by a significant loss of muscle mass/reductions in muscle fiber diameter and a decline in muscle force production. Given that skeletal muscle wasting can occur as early as after 3–5 days of disuse/unloading [6,7,8,9,10] and is associated with poorer health outcomes and survival rates in critically ill patients [11,12,13], disuse-induced muscle atrophy represents a vital issue to address. However, to date, no ideal treatment exists to combat disuse-induced loss of muscle mass and strength. Since disuse-related muscle atrophy primarily results from an imbalance between the rates of muscle protein synthesis and muscle proteolysis (the rate of protein degradation exceeds the rate of protein synthesis) [14,15], it is important to better understand underlying anabolic and catabolic signaling pathways in order to identify critical molecular targets for intervention. One such putative target is AMP-activated protein kinase (AMPK), a serine/threonine kinase that is able to switch on catabolic pathways involved in the generation of ATP while switching off anabolic pathways implicated in the ATP-consuming processes, including protein synthesis [16]. Indeed, it has been demonstrated that AMPK can suppress muscle protein synthesis via inhibition of the mammalian target of rapamycin complex 1 (mTORC1) [17,18,19] and promote muscle protein degradation by activation of FoxO and muscle-specific ubiquitin ligases muscle atrophy F-box (MAFbx) and muscle RING finger 1 (MuRF1) [20,21,22]. Evidence suggests that at the early period of mechanical unloading/disuse (0–3 days), AMPK activity (Thr172 phosphorylation) in postural soleus muscle is significantly reduced in both humans [23] and rats [24,25,26,27]. However, Cannavino et al. (2015) showed that 3-day unloading can lead to the upregulation of AMPK signaling (as assessed by acetyl-CoA carboxylase (ACC) phosphorylation) in mouse gastrocnemius muscle [28]. The discrepancy between the above-mentioned rat studies and the mouse study by Cannavino et al. (2015) could be attributed to profound metabolic differences between slow-type rat soleus muscle and fast-type mouse gastrocnemius muscle. Of note, a decrease in AMPK Thr172 phosphorylation in rat soleus muscle at the initial stage of muscle disuse is accompanied by the upregulation of mTORC1-signaling [24,26] and the maintenance of AMPK activity with AICAR pre-treatment is able to reduce mTORC1 activity to control levels after 1-day unloading [27]. Furthermore, increased mTORC1 activity observed in rat soleus at the initial stage (24 h) of unloading can lead to the upregulation of muscle-specific E3 ubiquitin ligases via nuclear export of HDAC5 [27]. Moreover, recent evidence suggests that mTORC1 activation in rat soleus muscle at the early stage (24 h) of mechanical unloading may contribute to the downregulation of translational capacity, as rapamycin treatment during 1-day hindlimb unloading prevents reductions in the key indicators of ribosome biogenesis (47S pre-rRNA and 18S+28S rRNAs) and partly attenuates a decrease in the rate of protein synthesis [29]. Possible causes of AMPK downregulation in rat soleus at the initial stages of mechanical unloading may include inactivity-induced accumulation of ATP [30] (a decrease in the AMP/ATP ratio) and/or glycogen accumulation [31] (binding of glycogen to AMPK can lead to reduced AMPK activity [32]). It is also important to note that at the later time points of mechanical unloading, AMPK Thr172 phosphorylation in rat soleus muscle either corresponds to control values (7-day unloading) [24] or goes above baseline levels (14-day unloading) [33,34]. At the same time, Egawa et al. (2015, 2018) demonstrated that AMPK activity in mouse soleus muscle is not affected in response to 14-day unloading [35,36]. Restoration of AMPK phosphorylation from day 0 to day 7 of unloading correlates well with the restoration of electromyographic activity of rat soleus during the course of hindlimb suspension [37]. It could possibly lead to an increase in muscle ATP consumption and subsequent restoration of AMPK phosphorylation. Thus, the level of AMPK (Thr 172) phosphorylation in the postural soleus muscle is significantly reduced during the first days of unloading, a period that precedes atrophy development. Therefore, we decided to conduct a study aimed at the maintenance of AMPK activity in rat soleus muscle during the first 3 days of mechanical unloading and examine possible anti-atrophic effects of such an approach. To this end, rats were pre-treated (for 7 days before unloading and during the first 3 days of unloading) with metformin, a widely accepted anti-diabetic agent that can specifically inhibit complex Ⅰ in the mitochondrial respiratory chain resulting in an increase in the AMP/ATP ratio in the cytoplasm and subsequent AMPK activation [38]. We hypothesized that the maintenance of AMPK activity with metformin pre-treatment would change the functioning of intracellular anabolic and catabolic processes and attenuate disuse-induced slow-to-fast fiber type transition as well as mitigate reductions in muscle weight, fiber size, and muscle strength.

## 2. Materials and Methods

### 2.1. Hindlimb Suspension

Mechanical unloading of rats was performed via rodent hindlimb suspension technique, as previously described [39,40]. In brief, the rat’s tail was wrapped with adhesive tape, and the animal was suspended by attaching a swivel hook to the metal bars on the top of the cage. The suspension height was adjusted to prevent the hindlimbs from touching any supporting surface while maintaining a suspension angle of approximately 35°. The rats were free to rotate in all directions within the cage and had access to food and water. The animals were checked daily for signs of tail lesions or discoloration.

### 2.2. Study Design

Male Wistar rats (2.5 months of age, 190–210 g), obtained from the Nursery for Laboratory Animals of the Institute of Bioorganic Chemistry of the RAS, Pushchino, Moscow region) were randomly divided into the following four groups (eight animals in each group): (1) control group (C), (2) control + 10-day metformin administration group (C+Met), (3) 7-day hindlimb suspension group (HS), and (4) a group of rats treated with metformin for 7 days before the onset of HS and during the first 3 days of 7-day HS (HS+Met). The rats were pre-treated with metformin for 7 days before HS and during the first 3 days of HS in order to prevent a decrease in AMPK activity, which is observed in rat soleus muscle during the first 3 days of hindlimb suspension [24,25]. The metformin-treated groups received 300 mg/kg/day of metformin dissolved in drinking water. The applied dose of metformin was selected based on previous studies [30,41,42,43]. Animals were housed in a temperature-controlled room on a 12:12-h light-dark cycle with food pellets and water provided ad libitum. On completion of the experiment, the rats were euthanized by isoflurane overdose followed by decapitation, and soleus muscles were rapidly removed and immediately frozen in liquid nitrogen or used for ex vivo measurements of muscle force.

### 2.3. Protein Extraction and Western Blot Analysis

A detailed description of protein extraction and Western blotting procedures can be found in our previous report [25,44].

In brief, muscle samples were loaded and separated on a 10% polyacrylamide gel, followed by transfer to a nitrocellulose membrane (Santa Cruz Biotechnology, Inc., Sanford, ME, USA, #sc-3724), after which membranes were incubated in a blocking buffer (TBS-T: 4% non-fat milk powder; Tris-buffered saline, pH 7.4; and 0.1% Tween 20). The membranes were then incubated with primary and secondary antibodies and washed in TBS-T. The primary antibodies used were phosphorylated Thr 183/172 AMPKα1/2 (1:1000, ABM, Richmond, BC, Canada, # Y408289), AMPKα (1:1000, Cell Signaling Technology, Danvers, MA, USA, #2532), phospho-AKT (Ser473) (1:1000, Cell Signaling Technology, Danvers, MA, USA, #4058), AKT (1:2000, Cell Signaling Technology, Danvers, MA, USA, #9272), phospho-GSK-3β (Ser 9) (1:1000, Cell Signaling Technology, Danvers, MA, USA, #9322), GSK-3β (1:1000, Cell Signaling Technology, Danvers, MA, USA, #12,456), phospho-4E-BP1 (Thr37/46) (1:1000, Cell Signaling Technology, Danvers, MA, USA, #2855), 4E-BP-1 (1:2000, Cell Signaling Technology, Danvers, MA, USA, #9452), phospho-p70S6K (Thr 389) (1:500; Santa Cruz Biotechnology, Santa Cruz, CA, USA, sc-11759), p70S6K (1:1000, Cell Signaling Technology, Danvers, MA, USA, #9202), phospho-FOXO3 (Ser253) (1:1000, Santa Cruz Biotechnology, USA, #sc-101683), and GAPDH (1:10,000, Applied Biological Materials Inc., Richmond, BC, Canada, # G041).

Then the membranes were incubated with HRP-conjugated secondary antibodies (1:30,000) to rabbit or mouse immunoglobulins from Santa Cruz Biotechnology, Santa Cruz, CA, USA (sc-2004). Protein bands were detected and analyzed using Clarity Western ECL Substrate (Bio-Rad Laboratories, Hercules, CA, USA) and C-DiGit Blot Scanner (LI-COR Biotechnology, Lincoln, NE, USA).

### 2.4. RNA Isolation and Agarose Gel Electrophoresis

Prior to RNA isolation, aliquots of frozen muscle tissue were weighed in order to calculate total RNA per mg of wet muscle tissue. RNA isolation and agarose gel electrophoresis were performed as reported previously [45,46]. RNA samples from equivalent amounts of tissue were run on gels in order to assess the content of 18S and 28S rRNAs. In brief, RNA electrophoresis was performed in 1.2% agarose gel with ethidium bromide staining in TBE buffer at 10 V/cm. The measurements of the 18S and 28S rRNA were performed by Gel Doc EZ imaging system (Bio-Rad Laboratories, Hercules, CA, USA). 

### 2.5. RT-PCR Analysis

RT-PCR analysis was performed as reported previously [25,47]. Briefly, total RNA extraction from frozen soleus muscle samples was provided using the RNeasy Micro Kit according to the manufacturer’s recommendations (Qiagen, Hilden, Germany). A total of 0.5 μg RNA was reverse-transcribed to cDNA using the RevertAid RT Reverse Transcription Kit (Thermo Scientific) according to the manufacturer’s instructions.

The compared samples were analyzed under similar conditions (template amounts, duration of PCR cycles). Real-time amplification was performed using SYBR Green I and the iQ5 multicolor real-time PCR detection system (Bio-Rad Laboratories, USA). PCR primers used for RNA analysis are shown in Table 1. The Pfaffl method was used to calculate relative gene expression [48,49]. RPL19, β-actin, and GAPDH were used as the housekeeping genes.

### 2.6. MyHC Immunostaining

Determination of the cross-sectional area (CSA) and the percentage of slow and fast muscle fibers can be found in our previous reports [44,50]. In brief, the soleus muscle sections were prepared with a Leica CM 1900 cryostat (Leica, Braunschweig) at −20 °C. Sections were incubated with primary antibodies MyHC I(β) slow (1:100 Sigma, St. Louis, MO, USA), MyHC fast (1:60, DSMZ) for 1 h at 37 °C and secondary antibodies Alexa Fluor 546 (1:1000; Molecular Probes, Waltham, MA, USA) for 60 min in the dark at room temperature. The soleus muscle sections were photographed with a Leica Q500MC fluorescent microscope at magnification ×20. Image analysis was processed by the ImageJ 1.52a software. At least 150 fibers were analyzed in each muscle sample (n = 8) for myofiber CSA measures, and at least 10 cross-sections per sample were examined to determine the percentage of different muscle fiber types in the sample (n = 8).

### 2.7. Ex Vivo Measurements of Soleus Muscle Contractility

Intact soleus muscles were carefully dissected from the hindlimb and allowed to equilibrate in oxygenated Ringer–Krebs solution (138 mM NaCl, 5 mM KCl, 1 mM NaH_2_PO_4_, 2 mM CaCl_2_, 2 mM MgCl_2_, 24 mM NaHCO_3_, 11 mM glucose). Contractile experiments were then performed as previously described [51]. Briefly, using silk sutures, soleus muscles were placed between two platinum electrodes at their optimal length, with one end tied to the arm of a dual-mode servomotor and the other end to a fixed, immovable hook. After single contractions, a tetanic isometric contraction test was performed. The muscle length was set at L_0_ and then stimulated with an electric field (40 Hz, 10V, for 3 s). Each tetanic contraction was followed by a 3-min rest to restore muscle contractility. The maximum force of tetanic contraction was recorded. Strength values obtained from 10 repetitions for each muscle were used. To normalize the parameters, the physiological muscle cross-section (CSA) was calculated [52]. Force measurements were carried out using an Aurora Scientific Dual Mode Lever System 305C-LR, with a data acquisition frequency of 10 kHz. Data processing was carried out using 615A Analysis Software Suite.

### 2.8. Statistical Analysis

Muscle weight and muscle strength data are shown as mean ± SEM. Morphological data, as well as Western blot and RT-PCR data, are shown as scatter dot plots indicating median values ± the minimum and the maximum. Sample medians are expressed as arbitrary units (a.u.). SigmaPlot 12.5 software package was used for statistical analysis. Since the normal distribution of the sample was not confirmed in all cases, a nonparametric Kruskal–Wallis test with Dunn’s multiple range test was applied. A *p* value less than 0.05 was regarded as statistically significant.

## 3. Results

### 3.1. Soleus Muscle Weight

Body weight in the C+Met, HS, and HS+Met groups was lower than that in the C group (Table 2). Normalized soleus muscle weight significantly decreased by 37% (*p* ˂ 0.05) in the HS group compared to the control group (Table 2). However, in the HS+Met group, normalized soleus muscle weight decreased only by 21% (*p* ˂ 0.05) compared to the control group (Table 2). Thus, metformin pre-treatment partly attenuated rat soleus muscle mass loss induced by 7-day mechanical unloading.

### 3.2. Cross-Sectional Area, Minimal Feret’s Diameter, and the Percentage of Slow and Fast Muscle Fibers

**Figure 1:**
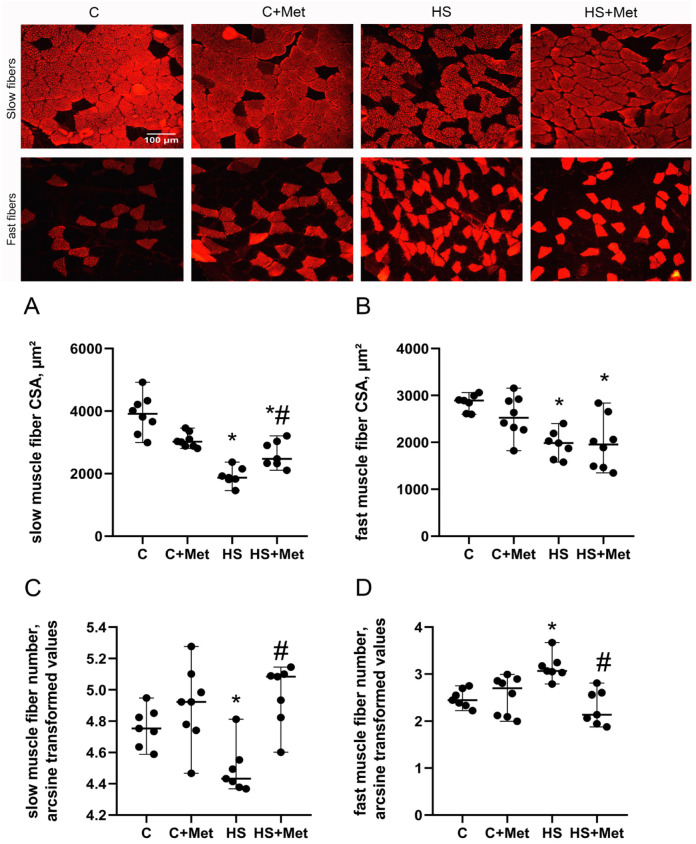
Cross-sectional area (CSA) of slow-type (**A**) and fast-type (**B**) fibers and the number of slow-type (**C**) and fast-type (**D**) fibers in rat soleus muscle. C, vivarium control, C+Met, vivarium control + metformin pre-treatment, HS, 7-day hindlimb suspension, HS+Met, metformin pre-treatment + 7-day hindlimb suspension. Scatter dot plots indicate median values, and the whiskers represent minimum and maximum values; n = 7–8/group. *: *p* < 0.05 vs. C, #: *p* < 0.05 vs. HS.

**Figure 2:**
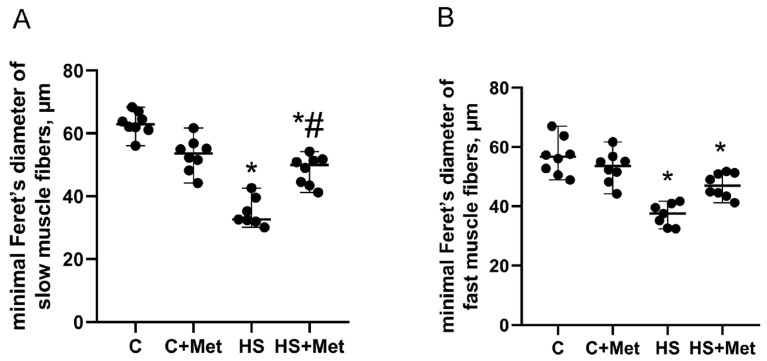
Minimal Feret’s diameters of slow-type (**A**) and fast-type (**B**) soleus muscle fibers. C, vivarium control, C+Met, vivarium control + metformin pre-treatment, HS, 7-day hindlimb suspension, HS+Met, metformin pre-treatment + 7-day hindlimb suspension. Scatter dot plots indicate median values, and the whiskers represent minimum and maximum values; n = 8/group. *: *p* < 0.05 vs. C, #: *p* < 0.05 vs. HS.

The cross-sectional area (CSA) of the soleus muscle slow-type fibers (expressing slow myosin heavy chain, MyHC I) significantly decreased by 52% (*p* ˂ 0.05) in the HS group compared to the control group (Figure 1). At the same time, the CSA of the soleus fast-type fibers (expressing fast isoforms of myosin heavy chain, MyHC II) significantly decreased by 31% (*p* ˂ 0.05) in the HS group compared to the control group (Figure 1). In the HS+Met group, the CSA of slow-type fibers increased by 15% relative to the HS group (*p* < 0.05), but the CSA of fast-type fibers remained at the levels observed in the HS group (Figure 1). The percentage of slow-type fibers significantly decreased by 13% after 7-day HS (*p* ˂ 0.05) vs. the C group, while the percentage of fast-type fibers significantly increased by 54% (*p* ˂ 0.05) in the HS group compared to the C group (Figure 1). In the HS+Met group, the percentage of both slow-type and fast-type fibers did not differ from the control group (Figure 1). Minimal Feret’s diameter significantly decreased in both slow-type and fast-type muscle fibers compared to the C group (Figure 2). Metformin pre-treatment partly prevented the HS-induced reduction in minimal Feret’s diameter of slow-type but not fast-type soleus muscle fibers (Figure 2).

### 3.3. Mechanical Properties of the Rat Soleus Muscle

The absolute maximum force of twitch and tetanic contractions of the isolated soleus in the HS group was reduced compared to the C group by 40% and 47% (*p* < 0.05), respectively (Table 3). In the HS+Met group, the absolute muscle twitch and tetanic tension significantly increased compared to the HS group but did not reach the control values (Table 3). At the same time, there were no significant differences in twitch, and tetanic tension normalized to muscle CSA between the groups (Table 3).

### 3.4. Anabolic and Catabolic Response of the Rat Soleus Muscle

**Figure 3:**
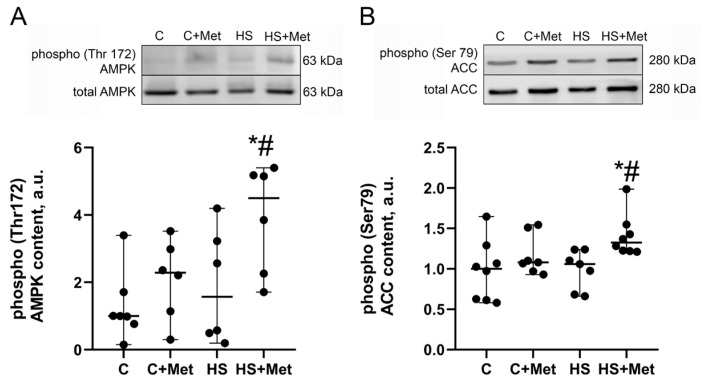
Effect of metformin pre-treatment on AMP-activated protein kinase (AMPK) (Thr172) phosphorylation (**A**) and acetyl-CoA carboxylase (ACC) (Ser79) phosphorylation (**B**) in rat soleus muscle. C, vivarium control, C+Met, vivarium control + metformin pre-treatment, HS, 7-day hindlimb suspension, HS+Met, metformin pre-treatment + 7-day hindlimb suspension. Scatter dot plots indicate median values, and the whiskers represent minimum and maximum values; n = 6–8/group. *: *p* < 0.05 vs. C, #: *p* < 0.05 vs. HS.

Metformin pre-treatment of the control rats induced an increase in AMPK (Thr 172) phosphorylation; however, the difference between the C and C-Met groups was not statistically significant (Figure 3A). Mechanical unloading for 7 had no significant effect on AMPK (Thr 172) phosphorylation in rat soleus muscle (Figure 3A). Metformin pre-treatment for 7 days before HS and during the first 3 days of unloading led to a significant increase (+350%, *p* < 0.05) in AMPK (Thr 172) phosphorylation compared to the control levels (Figure 3A). The overall pattern of acetyl-CoA carboxylase (ACC) phosphorylation was similar to that observed for AMPK (Figure 3B).

**Figure 4:**
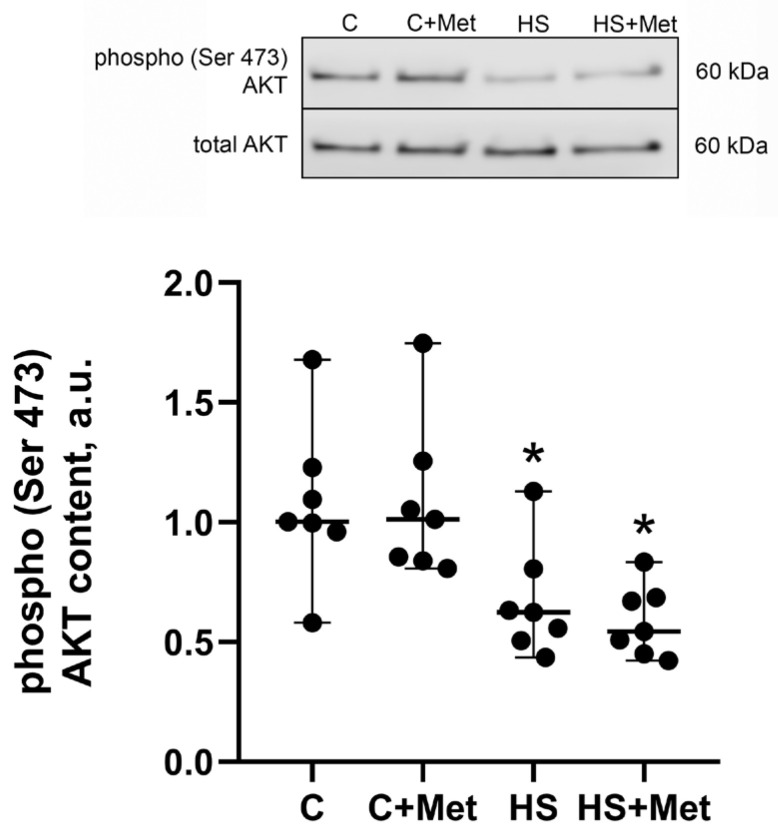
Effect of metformin pre-treatment on protein kinase B (AKT) (Ser473) phosphorylation in rat soleus muscle. C, vivarium control, C+Met, vivarium control + metformin pre-treatment, HS, 7-day hindlimb suspension, HS+Met, metformin pre-treatment + 7-day hindlimb suspension. Scatter dot plot indicates median values, and the whiskers represent minimum and maximum values; n = 7/group. *: *p* < 0.05 vs. C.

The levels of AKT/protein kinase B (Ser473) phosphorylation significantly decreased in rat soleus by 38% and 46% (*p* ˂ 0.05) in the HS and HS+Met groups, respectively, compared to the control group (Figure 4).

**Figure 5:**
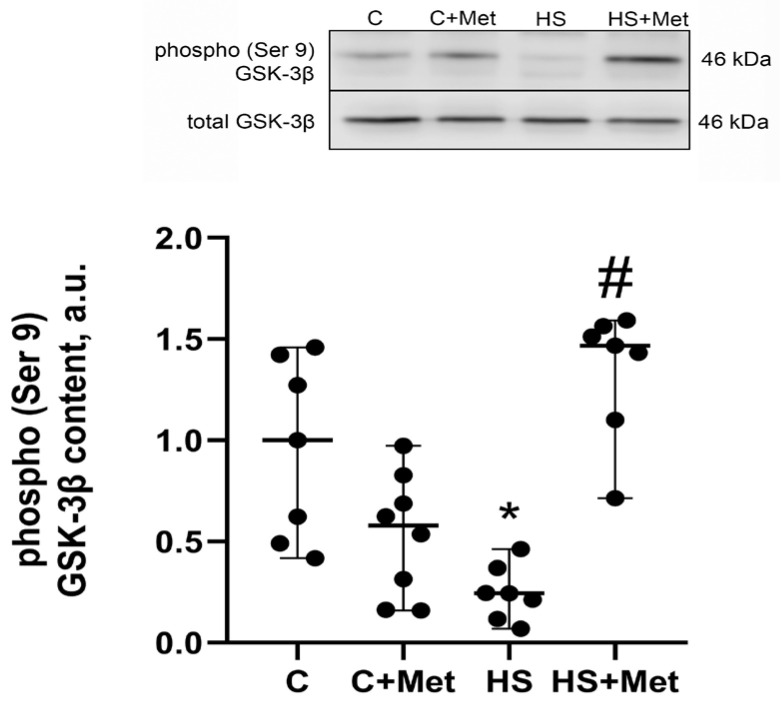
Effect of metformin pre-treatment on glycogen synthase kinase-3β (GSK-3β) (Ser 9) phosphorylation in rat soleus muscle. C, vivarium control, C+Met, vivarium control + metformin pre-treatment, HS, 7-day hindlimb suspension, HS+Met, metformin pre-treatment + 7-day hindlimb suspension. Data are shown as % of the C group. Scatter dot plot indicates median values, and the whiskers represent minimum and maximum values; n = 7–8/group. *: *p* < 0.05 vs. C, #: *p* < 0.05 vs. HS.

The level of glycogen synthase kinase-3β (GSK-3β) (Ser9) phosphorylation significantly decreased by 77% (*p* ˂ 0.05) in the HS group compared to the control group (Figure 5). However, in the HS+Met group, the level of GSK-3β (Ser9) phosphorylation did not differ from the control group and significantly increased by 124% (*p* ˂ 0.05) relative to the HS group (Figure 5).

**Figure 6:**
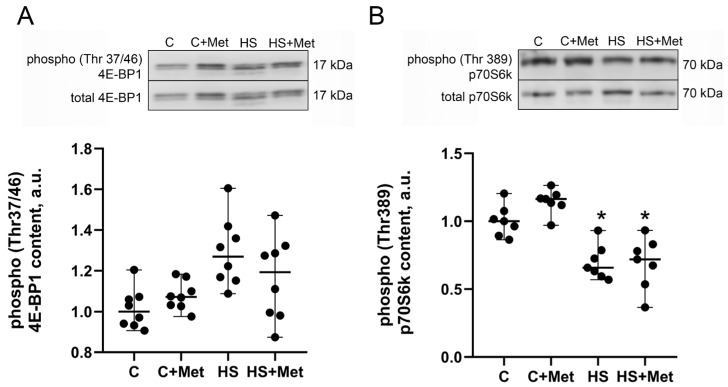
Effect of metformin pre-treatment on translation initiation factor 4E-binding protein 1 (4E-BP1) (Thr37/46) phosphorylation (**A**) and ribosomal protein S6 kinase beta-1 (p70S6K) (Thr389) phosphorylation (**B**) in rat soleus muscle. C, vivarium control, C+Met, vivarium control + metformin pre-treatment, HS, 7-day hindlimb suspension, HS+Met, metformin pre-treatment + 7-day hindlimb suspension. Scatter dot plots indicate median values, and the whiskers represent minimum and maximum values; n = 7–8/group. *: *p* < 0.05 vs. C.

One of the key signaling molecules regulating protein synthesis is protein kinase mTORC1 [53]. The phosphorylation level of translation initiation factor 4E-binding protein 1 (4E-BP1) (Thr37/46), a well-known mTORC1 substrate, did not significantly change across the groups (Figure 6A). The phosphorylation level of another mTORC1 substrate, ribosomal protein S6 kinase beta-1 (p70S6K) (Thr389), significantly decreased by 33% and 27% (*p* ˂ 0.05) in the HS and HS+Met groups, respectively, compared to the control group (Figure 6B).

**Figure 7:**
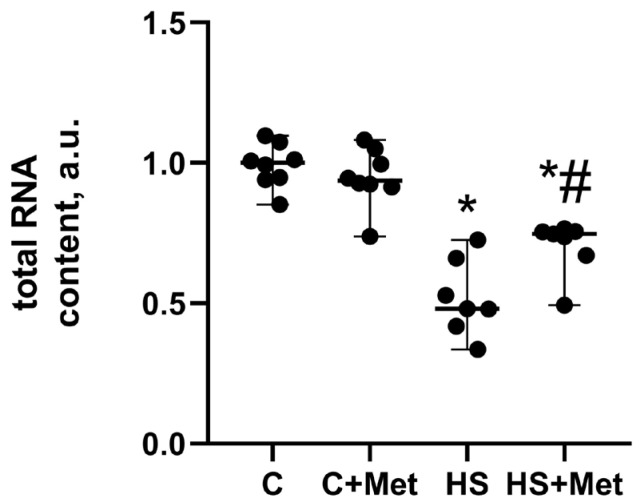
Effect of metformin pre-treatment on the total RNA content in rat soleus muscle. Total RNA content was normalized per mg of muscle tissue. C, vivarium control, C+Met, vivarium control + metformin pre-treatment, HS, 7-day hindlimb suspension, HS+Met, metformin pre-treatment + 7-day hindlimb suspension. Scatter dot plot indicates median values, and the whiskers represent minimum and maximum values; n = 7–8/group. *: *p* < 0.05 vs. C, #: *p* < 0.05 vs. HS.

Total RNA content in rat soleus significantly decreased by 52% (*p* < 0.05) in the HS group compared to the control group (Figure 7). In the HS+Met group, the total RNA content significantly increased by 27% (*p* < 0.05) relative to the HS group but remained significantly decreased by 25% (*p* < 0.05) compared to the control group (Figure 7).

**Figure 8:**
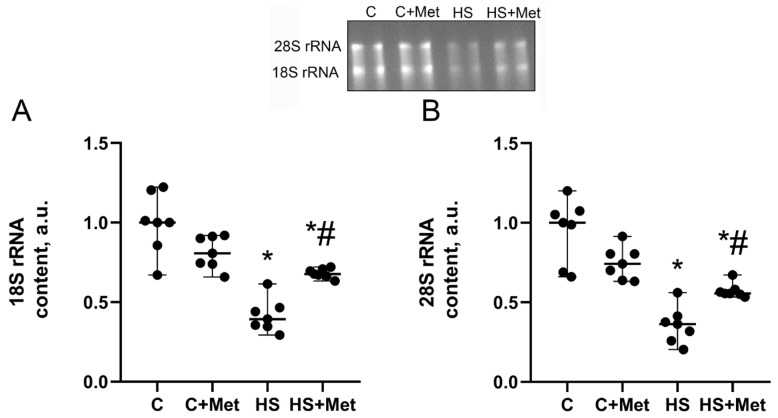
Effect of metformin pre-treatment on 18S rRNA content (**A**) and 28S rRNA content (**B**) in rat soleus muscle. C, vivarium control, C+Met, vivarium control + metformin pre-treatment, HS, 7-day hindlimb suspension, HS+Met, metformin pre-treatment + 7-day hindlimb suspension. Scatter dot plots indicate median values, and the whiskers represent minimum and maximum values; n = 7–8/group. *: *p* < 0.05 vs. C, #: *p* < 0.05 vs. HS.

The content of 18S and 28S rRNAs significantly decreased by 60% and 65% (*p* < 0.05), respectively, in the HS group compared to the control group (Figure 8). In the HS+Met group, the content of 18S and 28S rRNAs significantly increased by 28% and 21% (*p* < 0.05), respectively, relative to the HS group, but remained lower than that in the control group (Figure 8).

**Figure 9:**
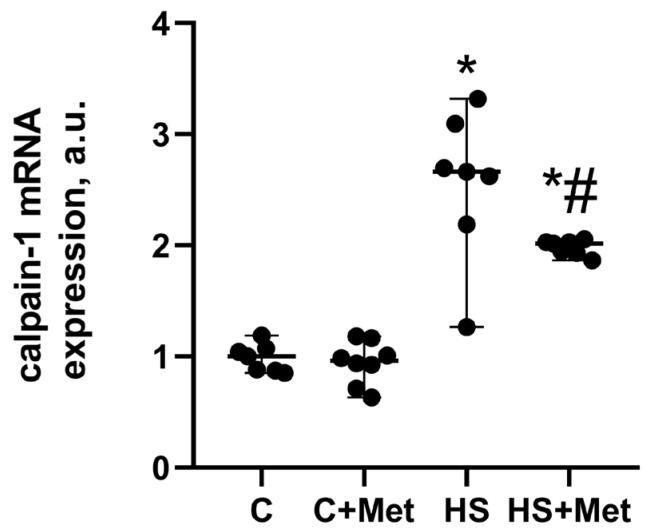
Effect of metformin pre-treatment on calpain-1 mRNA expression in rat soleus. C, vivarium control, C+Met, vivarium control + metformin pre-treatment, HS, 7-day hindlimb suspension, HS+Met, metformin pre-treatment + 7-day hindlimb suspension. Scatter dot plot indicates median values, and the whiskers represent minimum and maximum values; n = 7–8/group. *: *p* < 0.05 vs. C, #: *p* < 0.05 vs. HS.

Calpains, calcium-dependent proteases, may play an important role in muscle atrophy development [54]. In the present study, calpain-1 mRNA expression significantly increased by 166% (*p* < 0.05) in the HS group compared to the control group (Figure 9). In the HS+Met group, calpain-1 mRNA expression significantly decreased by 65% (*p* < 0.05) relative to the HS group but remained elevated compared to the control values (Figure 9).

**Figure 10:**
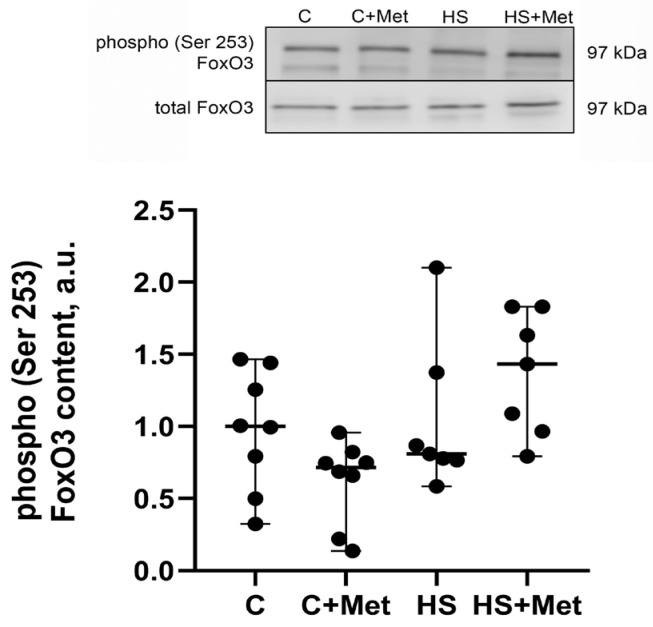
Effect of metformin pre-treatment on FoxO3 (Ser 253) phosphorylation in rat soleus muscle. C, vivarium control, C+Met, vivarium control + metformin pre-treatment, HS, 7-day hindlimb suspension, HS+Met, metformin pre-treatment + 7-day hindlimb suspension. Scatter dot plot indicates median values, and the whiskers represent minimum and maximum values; n = 7–8/group.

Our study did not reveal any significant differences in forkhead box O3 (FoxO3) (Ser253) phosphorylation across the groups (Figure 10).

**Figure 11:**
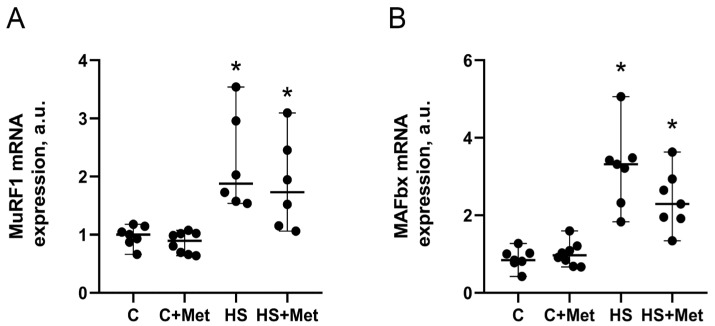
Effect of metformin pre-treatment on muscle RING finger 1 (MuRF1) (**A**) and muscle atrophy F-box (MAFbx/atrogin-1) (**B**) mRNA expression levels in rat soleus muscle. C, vivarium control, C+Met, vivarium control + metformin pre-treatment, HS, 7-day hindlimb suspension, HS+Met, metformin pre-treatment + 7-day hindlimb suspension. Scatter dot plots indicate median values, and the whiskers represent minimum and maximum values; n = 6–8/group. *: *p* < 0.05 vs. C.

The expression levels of muscle-specific E3 ubiquitin ligases, MuRF1 and MAFbx/atrogin-1, were significantly increased in rat soleus muscle in both HS and HS+Met groups compared to the control group (Figure 11).

**Figure 12:**
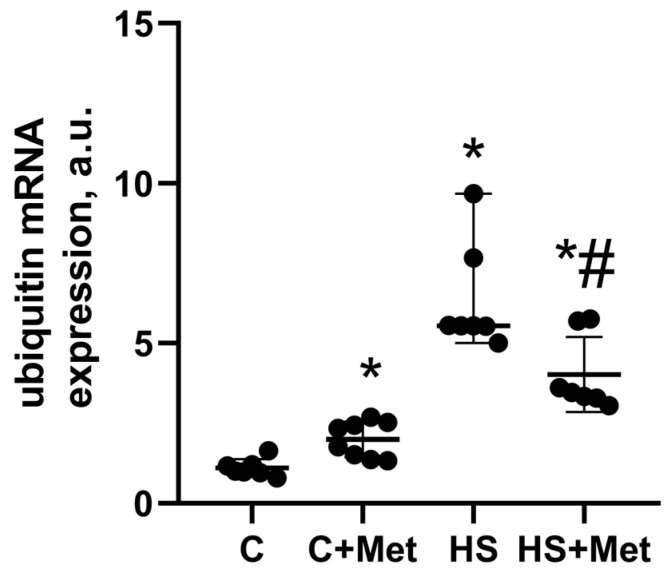
Effect of metformin pre-treatment on ubiquitin mRNA expression in rat soleus muscle. C, vivarium control, C+Met, vivarium control + metformin pre-treatment, HS, 7-day hindlimb suspension, HS+Met, metformin pre-treatment + 7-day hindlimb suspension. Scatter dot plot indicates median values, and the whiskers represent minimum and maximum values; n = 7–8/group. *: *p* < 0.05 vs. C, #: *p* < 0.05 vs. HS.

Ubiquitin mRNA expression significantly increased by 105%, 454%, and 247% (*p* < 0.05) in the C+Met, HS, and HS+Met groups, respectively, compared to the control group (Figure 12). In the HS+Met group, ubiquitin mRNA expression significantly decreased by 207% (*p* < 0.05) relative to the HS group (Figure 12).

## 4. Discussion

The aim of the present study was to find out if metformin pre-treatment would exert beneficial effects on rat soleus muscle morphology, force production, and intracellular signaling after 7-day mechanical unloading (hindlimb suspension). Rats were treated with metformin for 7 days before the onset of HS and during the first 3 days of 7-day HS in order to maintain the level of AMPK activity, which is known to be downregulated at the early stage of unloading/disuse in rat soleus muscle [24,25,26]. Our study demonstrated a protective capacity of metformin pre-treatment against disuse-induced loss of postural muscle mass and strength, reduced fiber size, and slow-to-fast fiber transition. These metformin-induced changes in rat soleus under disuse conditions were accompanied by significant alterations in several markers of anabolic and catabolic signaling pathways. However, it should be clearly stated that the dose and treatment duration used in the present study was not able to induce a statistically significant response in AMPK and ACC phosphorylation in rat soleus muscle in the C+Met group. Activation of the AMPK signaling pathway in rat soleus muscle was observed only in the HS+Met group.

Metformin, a widely prescribed anti-diabetic drug, is a known stimulator of AMPK activity in both isolated rat skeletal muscle [55] and in skeletal muscles of humans with type 2 diabetes [56]. According to existing literature, the effect of metformin on mammalian skeletal muscles remains controversial. On the one hand, it has been demonstrated that metformin administration can blunt muscle hypertrophy in response to resistance exercise in older subjects [57]. Furthermore, Kang et al. (2022) have recently shown that metformin administration (250 mg/kg, three times a week for four weeks) is able to induce an increase in myostatin mRNA expression and concomitant muscle fiber atrophy in mouse gastrocnemius but not tibialis anterior or extensor digitorum longus muscles [58]. In vitro studies suggested that metformin treatment is capable of promoting myotube atrophy via the AMPK–FoxO3a–HDAC6 axis [58]. On the other hand, a lot of studies showed the beneficial effects of metformin in conditions of muscle tissue damage or atrophy. Metformin has been shown to protect skeletal muscles from cardiotoxin-induced degeneration [59] and alleviate muscle wasting after burn injury by increasing the proliferation of satellite cells [60]. Hasan et al. (2019) have demonstrated that metformin administration can ameliorate obesity-induced rat soleus muscle atrophy, in part, via regulation of the PGC-1α-FoxO3 pathway [61]. Furthermore, metformin treatment has been shown to attenuate skeletal muscle atrophy caused by glutaredoxin-1 deficiency in mice [62] as well as increase muscle strength, improve muscle fiber membrane integrity, and diminish neuromuscular deficits in *mdx* mice [63]. Wang et al. (2022) have recently revealed that AMPK activation by metformin can suppress TGF-β1 overexpression and TGF-β1-induced Smad2/3 phosphorylation leading to reductions in myogenic contracture and myofibrosis induced by rat knee joint immobilization [64]. Recent evidence also suggests that metformin treatment may be conducive to the prevention of age-related sarcopenia by regulating lipid metabolism in skeletal muscle [65]. In addition, Petrocelli and colleagues (2021) reported that a dual treatment of metformin and leucine could improve skeletal muscle quality during disuse in aged mice by maintaining grip strength, soleus muscle force decrements, as well as by alleviating gastrocnemius collagen accumulation during hindlimb unloading [66].

In the present study, metformin pre-treatment partially preserved soleus muscle mass, absolute twitch and tetanic tension, and slow-type fiber diameter after 7-day HS. Furthermore, slow-to-fast fiber-type transformation was fully prevented in metformin-treated rats. These data are in favor of the concept that metformin can serve as a protective agent under conditions causing skeletal muscle wasting. Indeed, as described above, there is ample evidence that metformin administration can ameliorate skeletal muscle loss and function due to glutaredoxin-1 deficiency, obesity, or sarcopenia. One recent study showed that metformin administration during 3-day mechanical unloading was not able to prevent a reduction in rat soleus muscle mass but attenuated an increase in mRNA expression of some muscle atrophy markers [30]. It appears that the duration of metformin treatment in that study (3 days) was not long enough to induce changes leading to the full preservation of muscle proteins and, consequently, muscle mass. Moreover, metformin treatment of rats in parallel with one-week unloading does not seem sufficient to attenuate or prevent HS-induced reductions in skeletal muscle weight and fibers CSA, although such treatment is able to prevent slow-to-fast fiber transformation and upregulation of atrophic genes [67]. In contrast, the metformin treatment protocol used in the present study (7 days before the onset of unloading and during the first 3 days of one-week unloading) did attenuate soleus muscle atrophy (decreased soleus weight and slow-type CSA). Thus, treatment of rats with metformin well before the onset of mechanical unloading proves to be more beneficial in terms of soleus muscle preservation than concurrent metformin treatment. In the present study, 7-day HS did not induce any significant changes in AMPK activity in rat soleus muscle (as assessed by phosphorylation levels of AMPK Thr172 and ACC Ser 79), which is in line with a previously published paper [24]. AMPK activation that was observed in the HS-Met group did not affect Akt (Ser473) phosphorylation, FoxO3 (Ser253) phosphorylation, and mRNA expression of the atrophic genes (MuRF1 and MAFbx) compared to the HS group. However, metformin pre-treatment attenuated the unloading-induced upregulation of both calpain-1 and ubiquitin in rat soleus muscle. A significant upregulation of genes related to the ubiquitin–proteasome and calpain systems in rat soleus under disuse conditions is in agreement with a number of previously published reports [8,10,27,30,68].

Calpains are non-lysosomal cysteine proteases involved in skeletal muscle atrophy by cleavage of target proteins [69]. Ubiquitin is a regulatory protein that plays an important role in the ubiquitin–proteasome pathway as ubiquitin chains target proteins to the proteasome for degradation [70]. Thus, partial prevention of the increase in the levels of calpain-1 and ubiquitin mRNA expression in the HS+Met group could partly contribute to the protection of soleus muscle mass from HS-induced atrophy. Apart from catabolic processes, diminished anabolic signaling (i.e., pathways controlling protein synthesis) is known to contribute to disuse-induced skeletal muscle atrophy. Protein synthesis is determined by translational efficiency (the rate of protein synthesis per unit RNA) and translational capacity (the total ribosomal content per unit tissue) [71]. Translational efficiency largely depends upon mRNA translation initiation and hence mTORC1 activity. In the present study, 7-day HS resulted in a significant decrease in the phosphorylation status of p70S6K (a key mTORC1 substrate) in the soleus muscle compared to the control rats. This HS-related reduction in p70S6K Thr389 phosphorylation in rat soleus is in accord with previously published data [50,72,73,74,75,76]. At the same time, we did not observe any alterations in 4E-BP1 Thr37/46 phosphorylation in rat soleus muscle after HS. The exact cause of this phenomenon is not clear; however, possible explanations for a distinct response of p70S6K (Thr389) and 4E-BP1 (Thr37/46) phosphorylation to various conditions are discussed elsewhere in the literature [77,78,79,80,81]. Our study shows that metformin pre-treatment of rats that underwent 7-day HS (HS+Met group) has no effect on the phosphorylation of such markers of translational efficiency as p70S6K and 4E-BP1 in comparison to the HS group. However, metformin pre-treatment partly attenuated the unloading-induced reduction in the key markers of translational capacity, i.e., total RNA and 18S+28S rRNAs, in rat soleus muscle. A significant reduction in the markers of translational capacity in skeletal muscles under disuse conditions is in good agreement with previously published reports [9,50,82,83]. Furthermore, metformin administration fully prevented the HS-induced decrease in inhibitory Ser 9 phosphorylation of GSK-3β, a known negative regulator of protein synthesis [84]. Indeed, it has been recently demonstrated that GSK-3 inhibition during 7-day hindlimb unloading is able to attenuate reductions in both the markers of translational capacity and in vivo rates of muscle protein synthesis in rat soleus muscle [45]. A putative mechanism that could link GSK-3β activity with translational capacity is the inhibition of ribosome biogenesis via suppression of RNA polymerase I. Vincent and colleagues have shown (although in non-muscle cells) that GSK-3β is selectively enriched in nucleoli of RAS-transformed cells and is linked to the promoter region of the rDNA [85]. Moreover, these authors observed that GSK-3β inhibition is able to upregulate 45S, 18S, and 28S rRNA synthesis in RAS-transformed cells, confirming a repressive function for GSK-3β in rRNA biogenesis [85]. In addition, GSK-3 activity is associated with signaling pathways involved in the regulation of such important processes as degradation of myofibrillar proteins, mitochondrial biogenesis and myosin phenotype remodeling (slow MyHC expression) [84]. Thus, some beneficial effects of metformin pre-treatment on the unloaded rat soleus muscle, observed in our study, could be partly associated with the inhibition of GSK-3β activity via the maintenance of Ser9 inhibitory phosphorylation at the control levels. One possible mechanism linking metformin-induced AMPK activation and concomitant GSK-3β inhibition (prevention of the HS-induced decrease in GSK-3β Ser 9 phosphorylation), observed in the present study, could involve AMPK-dependent phosphorylation and activation of nitric oxide synthase (NOS), NO production and subsequent inhibition of GSK-3β activity via NO-dependent guanylate cyclase (GC)/cyclic guanosine monophosphate (cGMP)/PKG signaling pathway [86,87]. Indeed, it has been previously shown that AMPK activation leads to phosphorylation and activation of NOS (and hence NO production) in endothelial cells, myocytes [88,89] and in human skeletal muscle [90].

The results of the present study suggest a positive role in the maintenance/activation of AMPK activity (with metformin pre-treatment) in rat soleus muscle at the early stage of HS. However, previous studies by Egawa et al. (2015, 2016) demonstrated that reduction in soleus muscle weight in response to 14-day HS in transgenic mice that overexpress the muscle-specific dominant-negative mutant of AMPKα1 (AMPK-DN mice) was partly attenuated compared to wild-type mice [35,36]. This reduced AMPK activity (AMPK-DN mice) prevented HS-induced upregulation of the markers of the ubiquitin–proteasome system [35] but did not affect the transition of myosin heavy chain (MyHC) isoforms in response to unloading [36]. However, it should be noted that these studies were performed at later stages of mechanical unloading (14 days) compared to the present study, and the activity of the AMPK signaling pathway in rat soleus muscle can significantly differ at the early and later stages of mechanical unloading. It is also important to note that, in the present study, the expression levels of the key muscle-specific E3 ubiquitin ligases (MuRF1 and MAFbx) in the HS+Met group were upregulated, strongly suggesting that catabolic processes were still activated in rat soleus muscle by the 7th day of HS. Taking into account this fact and the above-mentioned studies, one possible hypothesis is that the metformin pre-treatment protocol used in the present study partially protects rat soleus muscle (as assessed by muscle weight and fiber CSA) between day 0 and day 7 of HS but may be inefficient for longer periods of HS.

There are several limitations in the current study that could be addressed in future research. First, the duration of the unloading in the present study was only 7 days. While the 7-day HS period is enough to induce significant alternations in intracellular signaling and subsequent skeletal muscle atrophy, it would be important to investigate whether the effect of metformin pre-treatment on skeletal muscles lasts longer than 7 days of mechanical unloading. Second, in the present study, only slow-type soleus muscle has been investigated. Although the “antigravity” soleus muscle is more susceptible to disuse/microgravity conditions than fast-type muscles, it would be interesting to investigate the effect of metformin pre-treatment on several fast-type muscles in future studies. Another limitation of the study is related to immunohistochemical analysis of muscle fibers. The results of the immunohistochemical analysis do not distinguish between different types of fast muscle fibers (IIa, IId/x, and IIb). Due to technical reasons, we were unable to perform an immunohistochemical myosin double/triple staining method for the identification of all fiber types in rat soleus muscle.

In summary, the present findings show that pre-treatment of rats with metformin (for 7 days before HS and during the first 3 days of one-week HS) significantly ameliorates HS-induced reductions in soleus muscle weight, slow-type fiber diameter, and absolute muscle strength. Metformin pre-treatment also fully prevented HS-related slow-to-fast fiber-type transformation in rat soleus muscle. These positive effects of metformin pre-treatment were accompanied by the partial rescue of translational capacity as well as by partial attenuation of increased mRNA expression of calpain-1 and ubiquitin.

## Figures and Tables

**Table 1:** Primers used for RT-PCR analysis.

Gene Description	Forward Primer	Reverse Primer
Calpain-1	5′-CATGGCTAAGAGCAGGAAGG-3′	5′-CGAAGTCTGCAGGTCTAGGG-3′
MAFbx	5′-CTACGATGTTGCAGCCAAGA-3′	5′-GGCAGTCGAGAAGTCCAGTC-3′
MuRF1	5′-GCCAATTTGGTGCTTTTTGT-3′	5′-AAATTCAGTCCTCTCCCCGT-3′
Ubiquitin	5′-CACCAAGAAGGTCAAACAGGA-3′	5′-GCAAGAACTTTATTCAAAGTGCAA-3′
RPL19	5′-GTACCCTTCCTCTTCCCTATGC-3′	5′-CAATGCCAACTCTCGTCAACAG-3′
Actb	5′-TCATGAAGTGTGACGTTGACATCC-3′	5′-GTAAAACGCAGCTCAGTAACAGTC-3′
Gapdh	5′-ACGGCAAGTTCAACGGCACAGTCAA-3′	5′-GCTTTCCAGAGGGGCCATCCACA-3′

**Table 2:** Changes in body weight and soleus muscle weight.

Group	Body Weight, g	Soleus Weight, mg	Soleus Weight/Body Weight, mg/g
C	271 ± 7.7	140 ± 3.2	0.52 ± 0.021
C+Met	215 ± 7 *	123 ± 5.5	0.57 ± 0.024
HS	204 ± 19 *	68 ± 3.5 *	0.33 ± 0.016 *
HS+Met	187 ± 10 *	77 ± 4 *#	0.41 ± 0.023 *#

Values are means ± SEM. C, vivarium control, C+Met, vivarium control + metformin pre-treatment, HS, 7-day hindlimb suspension, HS+Met, metformin pre-treatment + 7-day hindlimb suspension; n = 8/group. *: *p* < 0.05 vs. C, #: *p* < 0.05 vs. HS.

**Table 3:** Changes in the absolute and normalized twitch and tetanic tension in the isolated rat soleus muscle.

	C	C+Met	HS	HS+Met
Twitch tension, mN	104.8 ± 7.4	94.6 ± 4.4	59.4 ± 6.2 *	79.2 ± 7.1 *#
Twitch tension/CSA, mN/mm^2^	15.2 ± 1.2	15.9 ± 1	19 ± 2.5	18.2 ± 1.5
Tetanic tension, mN	718.6 ± 45.8	640.4 ± 38.9	385.5 ± 40.7 *	455.3 ± 37.7 *#
Tetanic tension/CSA, mN/mm^2^	103.6 ± 6.2	96.81 ± 16.3	121.1 ± 12.1	122.1 ± 10.9

Values are means ± SEM. C, vivarium control, C+Met, vivarium control + metformin pre-treatment, HS, 7-day hindlimb suspension, HS+Met, metformin pre-treatment + 7-day hindlimb suspension; n = 8/group. *: *p* < 0.05 vs. C, #: *p* < 0.05 vs. HS. CSA, muscle cross-sectional area.

## Data Availability

The data presented in the study are available upon reasonable request from the corresponding author.
